# Maternal stress, cord blood zinc and attention deficit hyperactivity disorder

**DOI:** 10.1038/s44184-025-00149-3

**Published:** 2025-08-07

**Authors:** Nagahide Takahashi, Tomoko Nishimura, Akemi Okumura, Toshiki Iwabuchi, Taeko Harada, Md Shafiur Rahman, Yoko Nomura, Jeffrey H. Newcorn, Kenji J. Tsuchiya

**Affiliations:** 1 Department of Developmental Disorders, National Center of Neurology and Psychiatry, Kodaira, Japan; 2 Research Center for Child Mental Development, Hamamatsu University School of Medicine, Hamamatsu, Japan; 3 United Graduate School of Child Development, The University of Osaka, Kanazawa University, Hamamatsu University School of Medicine, Chiba University and University of Fukui, Suita, Japan; 4 Queens College and Graduate Center, City University of New York, New York, NY USA; 5 Department of Psychiatry, Icahn School of Medicine at Mount Sinai, New York, NY USA; 6 Department of Child and Adolescent Psychiatry, Ichan School of Medicine at Mount Sinai, New York, NY USA

**Keywords:** Genetic association study, Neurodevelopmental disorders, ADHD, Risk factors

## Abstract

Zinc regulates dopaminergic signaling, and reduced serum zinc levels have been reported in individuals with ADHD. However, genetic associations between zinc and ADHD remain unclear. We examined this link using large-scale GWAS and molecular analyses across three cohorts: iPSYCH (14,584 ADHD cases and 22,492 controls), FAMHES (*n* = 1798), and the Hamamatsu Birth Cohort (*n* = 726). Two-sample Mendelian randomization revealed bidirectional associations between low serum zinc levels and ADHD diagnosis. Genetic correlation and polygenic risk score analyses supported this association. In the birth cohort, lower cord blood zinc were associated with higher ADHD symptom scores at ages 8–9. Zinc levels negatively correlated with IL-6 and maternal depressive symptoms. Directed acyclic graph analysis indicated that maternal stress increased IL-6, which reduced fetal zinc levels, linking to ADHD symptoms. These findings suggest low prenatal zinc may contribute to ADHD pathophysiology in genetically vulnerable children, potentially mediated by maternal stress and inflammation.

## Introduction

Attention Deficit Hyperactivity Disorder (ADHD) is the most prevalent neurodevelopmental disorder, beginning in childhood and often persisting across the lifespan. Both genetic and environmental factors are implicated in the disorder. Among several biomarkers^1^, reduced serum zinc levels have been repeatedly reported in subjects with ADHD^2^. Although the findings are not consistent across studies, a recent meta-analysis attributes this inconsistency to methodological issues such as small sample sizes, different measurements, and study designs^3^. ADHD is also associated with sleep disorders and altered melatonin production^4^. Zinc is known to regulate dopaminergic activity via its role in the production and metabolism of melatonin^5^, suggesting that zinc could be a potential target biomarker underlying the neurobiological mechanisms of ADHD. Despite its potential importance, to date, no genetic correlation or association between serum zinc levels and ADHD diagnosis has been explored.

Importantly, neuroinflammation has recently been proposed as contributing to the etiology of ADHD. In our previous study, we demonstrated that increased cytokines, such as IL-6, in cord bloods are associated with increased ADHD symptoms later in childhood^6^. Furthermore, elevated levels of pro-inflammatory cytokines are reported to reduce serum zinc^7^. Taken together, we may hypothesize that reduced levels of zinc may be involved in the development of ADHD in children in conjunction with neuroinflammation.

Perinatal stress is known to be an important mediator of child development^8^, including ADHD^9^. It is also considered to be a cause of perinatal neuroinflammation, such as increased levels of IL-6 in the brains of offspring^10^. Taken together, these data raise the possibility that zinc could have a role in the development of ADHD [in childhood] through its association with the inflammation that accompanies maternal perinatal stress.

Using multiple genetic and molecular biological analyses, this study aimed to investigate the relationship between serum zinc and ADHD in the context of neuroinflammation associated with maternal perinatal stress.

## Methods

### Subjects

Data from the Lundbeck Foundation Initiative for Integrative Psychiatric Research (iPSYCH) and the Psychiatric Genomics Consortium, the Queensland Institute of Medical Research (QIMR), the Fangchenggang Area Male Health Examination Survey (FAMHES) and the Hamamatsu Birth Cohort for Mothers and Children (HBC study) were analyzed.

### Mendelian randomization analyses

Two-sample Mendelian Randomization (MR) analyses with regression analyses were conducted using iPsych and QIMR with standard variant harmonization procedures (https://mrcieu.github.io/TwoSampleMR/). We used a *P* value threshold of 5 × 10^−8^ to select single nucleotide polymorphisms (SNPs) associated with serum zinc levels and ADHD diagnosis. For the main analysis we used MR Eggar and Inverse Variance Weighted (IVW) per exposure-outcome. Potential horizontal pleiotropy was evaluated using Cochrane’s Q statistic test. Further details are provided in the Supplementary Note 1.

### Genetic correlation analysis

Genetic correlations (rg) between serum levels of zinc and ADHD were calculated by LD (linkage disequilibrium) score regression using iPsych and FAMHES. Since the difference in the ethnic population between serum zinc and ADHD genome-wide association study (GWAS), we utilized the “Popcorn” program, which can calculate trans-ethnic genetic correlation.

### Polygenic risk score analysis

Polygenic risk score (PRS) for subjects in HBC study was generated by PRS-CSx^11^ using a iPSYCH ADHD GWAS study^12^ for ADHD-PRS and FAMHES zinc GWAS study^13^ for zinc-PRS. Genome-wide genotyping of subjects in HBC study was performed using the Japonica Array 2.0 platform. Detailed information about quality controls and imputation of HBC genotypng data were described in the Supplementary Note 1. The threshold for selecting SNPs was set at a *P*-value of 0.05. To account for population stratification, 4 principal components (PCs) calculated with PLINK 1.9 were used. The criteria for SNP clumping were *r*^2^ > 0.1 within a 2 Mb window. PRS scores were calculated with P-value thresholds at 0.05. Standardized PRS scores (mean = 0 and standard deviation = 1) were used for the analyses. Further details are provided in the Supplementary Methods section.

### Molecular analysis

Molecular analyses were performed to investigate the association between cord blood zinc levels and ADHD symptoms as assessed by the ADHD-RS in children aged 8-9 years. Serum zinc and IL-6 levels were measured by enzyme-linked immunosorbent assays (ELISA) according to the manufacturer’s protocol. Maternal stress was measured using the Edinburgh Postnatal Depression Scale (EPDS) two weeks after delivery. Analyses were limited to this subsample from the larger HBC cohort, in whom all variables of interest were available. 726 participants included in the molecular analyses had both cord blood samples (zinc and IL-6) and genome-wide genotyping data, ADHD symptom scores assessed at 8–9 years of age as well as maternal EPDS scores.

### Statistical analysis

All analyses were conducted using Stata 16 software (StataCorp. 2019. Stata Statistical Software: Release 16. College Station, TX: StataCorp LLC). Directed Acyclic Graph (DAG) analysis was used to account for the relationship between variables. Statistical significance was set at *P* < 0.05, and all tests were 2-tailed.

### Ethical considerations

The study protocol was approved by the Hamamatsu University School of Medicine and University Hospital Ethics Committee (Ref No. 20-233). Written informed consent was obtained from each caregiver (usually the mother) for her own and her children’s participation. This study followed the STrengthening the Reporting of OBservational studies in Epidemiology (STROBE) reporting guideline and STROBE-MR guideline.

## Results

### Characteristics of subjects

14,584 individuals with ADHD and 22,492 controls were gathered from iPSYCH and PGC data. GWAS data for serum zinc were obtained from the 2603 individuals in the QIMR cohort and the 1798 individuals in the FAMHES cohort. A total of 726 participants (373 males, 353 females) with both genotyping data and ADHD symptoms, measured using the parent form of ADHD-RS scale at 8-9 years old, cord blood zinc and iL-6 were included in the analysis. The study population used for each analysis is summarized in Table 1 and detailed sample characteristics of HBC study are described in the Supplementary Table 1.

**Table 1:** Summary of the cohorts used in the analyses

Analysis method	Cohort1	Outcome	Cohort2	Outcome
Mendelian Randomization	QIMR, *N* = 2603	Serum zinc	iPSYCH (*N* = 37526)	ADHD diagnosis
Genetic correlation	FAMHES, *N* = 1798	Serum zinc	iPSYCH (*N* = 37526)	ADHD diagnosis
Polygenic Risk Score analysis	iPSYCH (*N* = 37526)	ADHD diagnosis	HBC (*N* = 726)	ADHD symptoms
	FAMHES, *N* = 1798	Serum zinc	HBC (*N* = 726)	ADHD symptoms

*QIMR* Queensland Institute of Medical Research, *iPSCH* Integrative Psychiatric Research, *FAMHES* the Fangchenggang Area Male Health Examination Survey, *ADHD* attention deficit hyperactivity disorder, *HBC* Hamamatsu Birth Cohort for Mothers and Children.

### Bidirectional association between zinc and ADHD

A bidirectional association was observed between low serum zinc levels and ADHD diagnosis. In the analysis of serum zinc to ADHD diagnosis, the association was significant (MR-Eggar beta [SE] = −0.049[0.009], *P* = 2.462 × 10^−8^ and IVW beta [SE] = −0.018[0.035], *P* = 2.405 × 10^−7^). The association of ADHD diagnosis to serum zinc was also significant (MR-Eggar beta [SE] = −0.965[0.238], *P* = 8.839 × 10^−5,^ IVW beta [SE] = −0.093[0.003], *P* = 7.619 × 10^−4^).

### Genetic correlation and PRS analyses

Genetic correlation analysis showed that serum zinc levels and ADHD diagnosis are genetically correlated (*r*_g_[SE] = −0.218 [0.004], *P* < 0.001). PRS analysis showed that Zinc-PRS was negatively associated with serum zinc (beta [SE] = −0.841[0.011], *P* < 0.001) and ADHD symptoms in children (beta [SE] = −0.484[0.033], *P* < 0.001). ADHD-PRS was negatively associated with serum zinc (beta [SE] = −2.036[0.796], *P* = 0.011) and ADHD symptoms in children as previously reported (beta [SE] = −0.907 [0.005], *P* < 0.001)^14^.

**Fig. 1:**
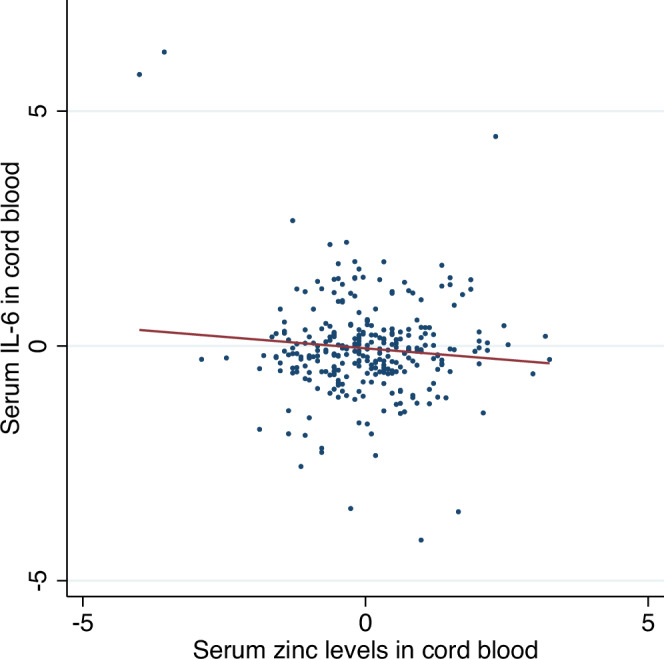
Correlation analysis between serum zinc and IL-6 in cord blood. Red line indicates best fitted line in regression analysis.

### Cord Blood Zinc and ADHD Symptoms

**Fig. 2:**
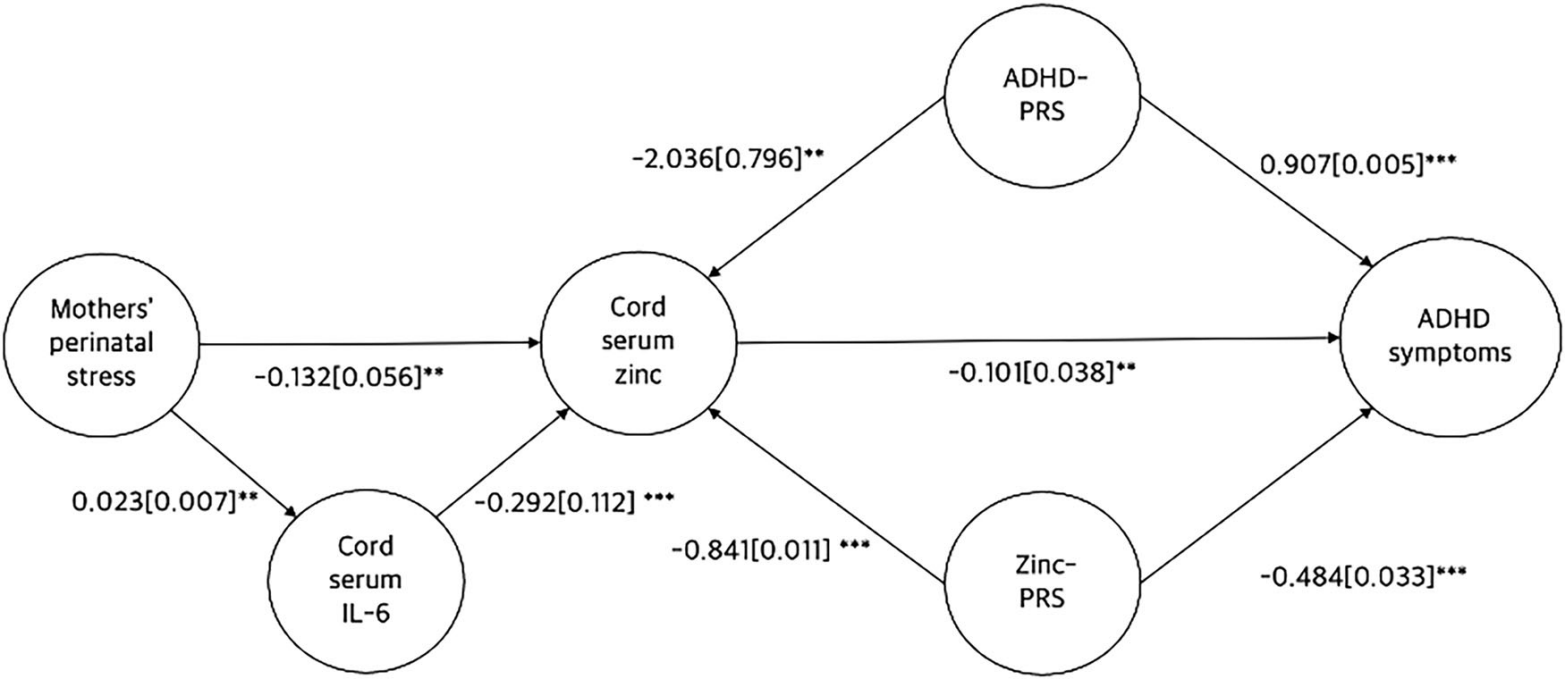
Directed acyclic graph between mother’s perinatal stress, serum zinc and A DHD symptoms. Numbers represents standardized beta by regression analysis. Numbers in [] represents standard error. *P < 0.05, **P < 0.01, ***P < 0.001.

Serum zinc levels were negatively associated with ADHD symptoms in children at age 8–9 (*r*[SE] = −0.101[0.038], *P* = 0.004). Serum zinc levels were also negatively correlated with IL-6 levels (*r*[SE] = −0.292[0.112], *P* < 0.001) (Figs. 1, 2). To address the potential influence of outliers, we conducted a sensitivity analysis excluding values above the 95th percentile for IL-6. The negative association between IL-6 and cord blood zinc remained statistically significant (β = −2.99, SE = 1.22, *p* = 0.015), indicating the robustness of the finding. We also conducted a regression analysis adjusting for maternal age, education level, and prenatal antidepressant medication use. The association between cord blood zinc levels and ADHD-RS scores remained significant (β = −0.102, SE = 0.038, *p* = 0.008), further supporting the robustness of the finding.

### Maternal Stress, Inflammation, and Zinc Levels

Mothers’ perinatal stress level as measured by the total EPDS score were negatively correlated with serum zinc levels (*r*[SE] = −0.132[0.056], *P* = 0.004). DAG analysis showed that maternal stress affects serum zinc levels via modulation of IL-6 levels. Reduced zinc levels, in turn, were associated with increased ADHD symptoms in children aged 8–9 (Fig. 2).

## Discussion

The present study showed that low serum zinc levels in cord blood are genetically and biologically associated with ADHD diagnosis and symptoms at age 8, possibly induced by maternal perinatal stress. Furthermore, our data proposes that IL-6 is one of the key molecules to connect maternal stress and zinc in the mechanism of ADHD pathophysiology. While zinc is known to influence dopaminergic signaling pathways, our genetic analyses did not specifically focus on dopamine-related SNPs. Instead, we employed genome-wide polygenic risk scores and Mendelian randomization based on large-scale GWAS summary statistics for ADHD and serum zinc. Multiple genetic analyses indicate a bidirectional relationship between genetic risk for ADHD and serum zinc levels. These findings suggest that lower serum zinc levels in cord blood may act as a biological vulnerability factor for the development of ADHD symptoms in genetically susceptible individuals. Although this raises the possibility that prenatal zinc supplementation could be beneficial, interventional studies are needed to test this hypothesis^15^.

A few limitations should be considered in this study. First, we used the EPDS score two weeks after delivery as a proxy for perinatal stress among pregnant individuals. Different measures of stress could have strengthened the validity of the construct. Second, although our model is statistically robust, the biological validity should be confirmed experimentally. The present findings should be replicated in independent cohorts and the benefit of zinc supplements should be examined in biological model systems (e.g., maternal-immune-activation models). Third, although we restricted our analyses to participants with complete data on cord blood zinc and IL-6 levels, ADHD symptoms, and genome-wide genotyping (*n* = 726), this subset may not be fully representative of the entire cohort. Therefore, selection bias cannot be entirely ruled out. Finally, the PRS construction was based on GWAS summary statistics from different ethnic backgrounds (European and Chinese), and while we used trans-ethnic methods (PRS-CSx), the generalizability to Japanese populations may still be limited.

## Supplementary information

Supplementary information

## Data Availability

The data generated for this study are subject to the following licenses/restrictions: Privacy and Confidentiality of Participants. Requests to access these datasets should be directed to N.T., nagahide.takahashi@ncnp.go.jp.
