# Supplementary material for: Maternal stress, cord blood zinc and attention deficit hyperactivity disorder
Source: Npj Ment Health Res. 2025 Aug 7;4:36. doi: 10.1038/s44184-025-00149-3 (PMC12331919; doi:10.1038/s44184-025-00149-3)
Supplement: Supplementary file 1 — Supplementary information [file 44184_2025_149_MOESM1_ESM.docx]

**Supplementary materials**

**Supplementary Note 1**

Study cohorts

Four study cohorts were used for the analysis.

Data from 1258 children was obtained from ongoing longitudinal cohort HBC Study.

The summary data for ADHD diagnosis was obtained from a GWAS study that included a population-based cohort collected by the Lundbeck Foundation Initiative for Integrative Psychiatric Research (iPSYCH), as well as from 11 European, North American, and Chinese cohorts aggregated by the Psychiatric Genomics Consortium (PGC). This summary data was used for mendelian randomization (MR), Linkage disequilibrium score-based genetic correlation (LDSC) and polygenic risk score (PRS) analyses.

The summary data for serum zinc was obtained a GWAS study that included 2603 individuals from the Queensland Institute of Medical Research (QIMR). This summary data was used for MR and GC analyses.

The summary data for serum zinc was obtained from a GWAS study that included 1798 Chinese males aged 17–88 years old. These individuals were enrolled from the Fangchenggang Area Male Health and Examination Survey (FAMHES). This summary data was used to calculate PRS in subjects from Hamamatsu birth cohort for mother and children (HBC study).

**Measurement**

ADHD symptoms in HBC study were assessed through interview by trained psychologists at 8-9 years old using the Japanese version of the ADHD-Rating Scale (ADHD-RS), which has been previously validated and proven reliable^21^. The ADHD-RS includes two sub-scales: inattention (nine items) and hyperactivity-impulsivity (nine items). Each item was rated on Likert scale ranging from 0 (never or rarely) to 3 (very often). Trained interviewers conducted the evaluations.

Serum zinc and IL-6 levels in subjects from HBC study were measured as follows: Ten ml of umbilical cord blood was collected from children immediately after delivery via venipuncture of the umbilical vein. The samples were kept at room temperature for 30 minutes after collection and then centrifuged at 3500 rpm for 10 minutes, divided in 200 μl aliquots and stored at -80°C until analysis. The concentration of zinc was measured by inductively coupled plasma mass spectrometry (ICP-MS) and IL-6 was measured by enzyme-linked immunosorbent assays (ELISA) according to the manufacturer’s protocol. All samples were analyzed in triplicate, and the mean score was used for analysis.

ADHD diagnosis was made according to ICD10 (F90.0) in PSYCH cohort.

Genotyping

Genotyping of participants in the HBC cohort was conducted using the Japonica array 2.0 (500K). Quality controls were applied to retain Single Nucleotide Polymorphisms (SNPs). Subjects with the following criteria: missing data for SNP < 0.02, pairwise Identity-By-Descent (IBD) < 0.2, SNP Hardy Weinberg equilibrium of P > 10-6 and minor allele frequency > 0.01. A total of 76 subjects were removed from the analysis as "related subjects" based on IBD analysis.

Genotype imputation was performed using BEAGLE 5.0 with JPK 4.1 as references, as described previously (Takahashi et al., 2020). SNPs with an imputation INFO score < 0.8 were excluded. SNPs located within the MHC region were also excluded due to high linkage equilibrium in this region. The number of SNPs analyzed for PRS was 5,606,655.

PRS calculation

PRS was generated by PRS-CSx (https://github.com/getian107/PRScsx) which enables to calculate PRS using GWAS summary data obtained from cohorts with different ethnicity. Using a recent iPSYCH GWAS study for ADHD (Demontis et al., 2019) and a FAMHES GWAS study conducted in China (Yang et al., 2022) were used as discovery cohorts. The summary GWAS data of ADHD was obtained from the Psychiatric Genomics Consortium (https://www.med.unc.edu/pgc/) and the summary data of serum zinc were obtained from a GWAS catalog (https://www.ebi.ac.uk/gwas/publications/35501403). The threshold for selecting SNPs was set at a P-value of 0.05.

To account for population stratification, 4 principal components (PCs) calculated with PLINK 1.9 were used. The criteria for SNP clumping were r2 > 0.1 within a 2Mb window. PRS scores were calculated with P-value thresholds at 0.05. Standardized PRS scores (mean = 0 and standard deviation = 1) were used for the analyses. Covariates included 4 principal components, social responsive scales 2 (SRS-2), which may influence ADHD-RS scores. and gender.

Genetic correlation analysis

Genetic correlations (r_g_) between serum zinc levels and ADHD diagnosis were calculated using the Popcorn (https://dougspeed.com). The summary data of serum zinc ([GCST90101875]) and ADHD ([GCST007543]) was obtained from the GWAS catalog (https://www.ebi.ac.uk/gwas/home).

Mendelian randomization

Two-sample MR analyses with regression analyses were conducted, with standard variant harmonization procedures (https://mrcieu.github.io/TwoSampleMR/)^23^. We used a P value threshold of 5 × 10^−８^ to select SNPs. For the main analysis we used MR Eggar and Inverse Variance Weighted (IVW) per exposure-outcome. Potential horizontal pleiotropy was evaluated using Cochrane’s Q statistic test.

**Supplementary Table 1**

**Characteristics of subjects from Hamamatsu birth cohort for mother and children (HBC study).**

| Characteristics | N or mean(SD) |
| --- | --- |
| Sex (male/female) | 373/353 |
| Maternal Age at Birth (years) | 31.9 (5.0) |
| Maternal Education (12yrs> / 12yrs <) | 698/28 |
| Maternal Antidepressant Use (Yes/No) | 11/715 |
